# Reviewers acknowledgement

**DOI:** 10.1002/2211-5463.70168

**Published:** 2025-12-01

The editors of *FEBS Open Bio* would like to thank all those who have given their time and expertise to review articles submitted for publication in Volume 15. Although *FEBS Open Bio* does not seek to judge the importance of submissions, our reviewers carefully scrutinize the experimental design and results of all papers, and challenge authors about their conclusions.

The names of these reviewers are listed below; we apologize if we have inadvertently omitted anyone.

Wade Abbott

Ahmed Abd El Wahed

Tadayuki Akagi

Bunyamin Akgul

Yunus Akkoç

Matthew Alexander

Yaaser Q. Almulaiky

Warner Alpizar

Alexey Amunts

Bogi Andersen

Mauricio Andino

David Andreu Martínez

Abdel Aouacheria

Munehito Arai

Satoshi Arai

Alexandra Araújo

Mariela Arias

Ami Aronheim

Avraham Ashkenazi

Yuji Atsuta

Ibrahim E. Awad

Raheela Awais

Takuya Azami

Jingkun Bai

Darren Baker

Aaqib Zaffar Banday

Meenakshi Banerjee

Thomas R. M. Barends

János Barna

Jiri Bartek

Alexander Bartelt

Brendan Battersby

Thomas Bauer

Ivan Bedzhov

Nitin Sai Beesabathuni

Angéla Békési

Brian Belardi

Georgios N. Belibasakis

Brendan Bell

Dhanush Bellapu

Giulia Bertolin

Sofia‐Iris Bibli

Vladimir Bilim

Claudia Binda

Bastien Bissaro

Clement Blanchet

Sara Blumer‐Schuette

Jens Bohne

Amelie Bonnet‐Garnier

Usa Boonyuen

Imre M. Boros

Kakoli Bose

Piotr Bragoszewski

Alexandra Carolyn Brand

Saverio Brogna

Andrei Budanov

Timothy Bugg

Olga Y. Burenina

Rebecca A. B. Burton

Loredana Bury

Laura Calvo

Marco Cammarata

Zachary Campbell

Adrian Canizalez‐Roman

David Cannella

Ruben Carbonell

Luiz Pedro Carvalho

David Castilla‐Casadiego

Luiz Augusto Cauz‐Santos

Ugo Cavallaro

Nathalie Cella

Jobichen Chacko

Rima Chakaroun

Krishnendu Chakraborty

Joydeep Chakraborty

Prasenjit Chakraborty

KuiMing Chan

Prabal K. Chatterjee

Mingyi Chen

Bill Cheng

Hsiang Cheng Chi

Gabriela Chiosis

Laurie Comstock

Tiago Cordeiro

I.J. Correia

Fasseli Coulibaly

Soren Zachary Coulson

Haissi Cui

Meng Cui

Zoran Culig

Colm Cunningham

William Curtis Hines

Virginie Dangles‐Marie

Iñaki de Diego Martinez

Luitzen de Jong

Erick De la Cruz‐Hernandez

Daniela De Zio

Marta De Zotti

Michael Decker

Nima Dehdilani

Maurizio Del Poeta

Tugce Demirel‐Yalciner

Sven Dennerlein

Sanket Desai

Gary Desir

Bernard Dieny

Roberto Dinami

Dayna Dreger

Cheng Du

M. Beatriz Durán‐Alonso

Roshan Dutta

H. Atakan Ekiz

Caglar Elbuken

Christoph Engel

Margarida Fardilha

Romana Fato

Nadia Fattahi

Valwynne Faulkner

Lorenzo Favaro

Fabrizio Febo

Alessandra Ferlini

Rafael Fernández‐Chacón

M. Rohan Fernando

Michael Fromm

Koichi Fujisawa

Naonobu Fujita

Fun Man Fung

Bijaya Gaire

Panagiotis Galanos

Silpa Gampala

José A. Gavira

Róna Gergely

Eduardo Hideo Gilglioni

Ignacio Giménez

Rafael L. Giner Arroyo

Griet Glorieux

Jun Gojobori

Tereza Golias

Rocio Gomez

F. Xavier Gomis‐Ruth

Vellore Kannan Gopinath

Kathleen L. Gould

Shivansh Goyal

Aleksander Grabiec

Thomas Grewal

Giovanna Grimaldi

Paolo Grumati

Jianguo Gu

Laura Gutiérrez

Joshua J. Hamey

Roslida Abd Hamid

Toshikatsu Hanada

Nicholas Hand

Liang Hao

Md Azizul Haque

Tibor Harkany

Pirkko L. Härkönen

Patrick Harte

Anna‐Maria Hartmann

Junya Hasegawa

Zhao He

Femke Heindryckx

László Henn

Juan A. Hermoso

Felix Hernandez

Anna Hernandez‐Prat

Hayato Hirai

Rita Hirmondó

Seiji Hitoshi

David J. Hodson

Wanjin Hong

Su‐Hyung Hong

Yaoqin Hong

Mai Horiuchi

Gabriel Hornink

Li Hou

Susan Ellen Howlet

Jinghan Huang

Robert Joseph Huber

Ted R. Hupp

Julia Iermak

Luigi Ippolito

Ismail Hassan Ismail

Lars J. C. Jeuken

Xinwei Jiao

Urja Joshi

Alice Jouneau

Vanda Juranic Lisnic

Suhas Kadam

Krisztina Káldi

Kenya Kamimura

Ioannis Kanakis

Yannis Karamanos

Evgenia (Jenny) Karousou

Maja Katalinic

Fuminori Kawabata

Maria Kawalec

Robert Kay

Kenneth Keiler

Douglas B. Kell

Abdul Waheed Khan

Satish Khurana

William David Kim

Hye Kwon Kim

Barbara Klajnert‐Maculewicz

Jörg Kleeff

Melvin E. Klegerman

Miroslav Kloz

Tomo Kondo

Christos K. Kontos

Zrinka Kovarik

Oliver H. Krämer

Akihiro Kuma

Janesh Kumar

Samvid Kurlekar

Maximilian Lackner

Piotr Ladyzynski

Ming‐Chih Lai

Otmane Lamrabet

Douglas Laurents

Maria Cristina Lavagnolo

Inna N. Lavrik

Sylvia Emmanuelle Le Dévédec

David S. Leake

Hsin‐Chen Lee

Hyemin Lee

Sangjoon Lee

Alexander Leitner

Gaetano Leto

François Letourneur

Xiaoyan Li

Zeyuan Li

Guocai Li

Ya Chee Lim

Audrey Lin

Yuxin Lin

Zoltan Lipinszki

Jun Liu

Meilian Liu

Enza Lonardo

Montanaro Lorenzo

Peter Low

Juan Pablo Luaces

Peng Luo

Alex Lyakhovich

Niamh Lynam‐Lennon

Zack Ma

Vicky E. MacRae

Madathiparambil Gopalakrishnan Madanan

Tadao Maeda

Huanghao Mai

Michal Maj

Mija Marinković

Jonathan Martinez‐Fabregas

Felipe Martinez‐Pastor

Camille Mary

Antonio Mas

Ivica Matak

Michiya Matsusaki

Michael G. Mauk

Jeffrey McFarlane

Bailey McGuire

Jared M. McLendon

Gracjan Michlewski

Eduardo Rodriguez de San Miguel

Arpad Mike

Aleksandra Milewska Milewska

Heather Bennett Miller

Tom Misteli

Ahmed Mohammed

Gianluca Molla

Javier Mora

Yusuke V. Morimoto

Masahiro Morita

Cedric Moro

Simone Morra

Lorenzo Mortara

Aristidis Moustakas

Gabriele Multhoff

Sebastian Munck

Roberto Munita

Raphael Munoz

Vladimir Muzykantov

Reetoja Nag

Istvan Nagy

So Nakagawa

Yusuke Nakamichi

Junichi Nakayama

Sangeeta Nath

Matthias Nees

Sven Niklander

Natasa Nikolic

Nikolay Ninov

Hafumi Nishi

Guoqi Niu

Suegene Noh

Prapanca Nugraha

Kinga Nyíri

Maria Marjorette O Peña

Dominik Oberthuer

Mikolaj Ogrodnik

Mina Okochi

Mitsuru Okuwaki

Abraham Oluwole

Eugene Boon Beng Ong

Ana Orive‐Ramos

Enrique Ostria

Petar Ozretić

Nigel Page

Erwann Pain

Martina Palomino‐Schätzlein

Tibor Pankotai

Emilio Parisini

Sung Taek Park

Venkatachalam Parvathi

Maria Rosalia Pasca

Eszter Patakiné Várkonyi

Nick Peake

Alessio Peracchi

Arsen Petrovic

Thi‐Minh‐Hue Pham

Helen Philippou

Lai Yee Phoon

Paolo Pinton

Sergej Pirkmajer

Jelena J. Podgorac

Lejla Pojskic

Indira D. Pokkunuri

William J. Polacheck

Carlo Polidori

Marta Popovic

Matthew Pratt

Catherine Price

Monika Proszkowiec‐Weglarz

Timothy Pullen

Tanel Punga

Anđela Pustak

Sai Prasad Pydi

Mirza Muhammad Fahd Qadir

Ayyalusamy Ramamoorthy

Jian‐Hua Ran

Pablo Ranea‐Robles

Mee‐Ra Rhyu

Jan Riemer

Michael L. Risner

Mark Roberts

Vicente Roca Agujetas

Stéphane Roche

Cristina Rodríguez

Kyle Rohde

Alexander Romanishin

Maria Zelinda Romano

Antonio Rosato

Giovanna Rossi Marquez

Barak Rotblat

Luca Roz

Vicenç Ruiz de Porras

Wojciech Rypniewski

Andrey Rzhetsky

Silvia Sacchi

Kota Saito

Takeshi Sakai

Kimitoshi Sakamoto

Walter Sandter

Carolina Sanhueza

Victoria Rossmary Santacruz Oviedo

Takahiro Sato

Takaaki Sato

Herbert Sauro

Jon R. Sayers

Martin Schepelmann

Martin Schön

Gerrit Schut

Darcie Seachrist

Jomon Sebastian

Serif Senturk

Guy Servant

Yuji Seta

Katie Shannon

Rohit Sharma

Adam Sharp

Ao Shi

Osamu Shimozato

Tsuyoshi Shirai

Toshihiko Shiraishi

Sarah C. Shuck

Elena Shuyskaya

Cristina Silva Pereira

Tatiana de Almeida Simao

Agneta Simionescu

Tom J. Simpson

Brijesh Kumar Singh

William I. Sivitz

Vassiliki T. Skamnaki

Margaretha Agnes Skowron

Chad Slawson

Michel Sliwa

Cristian Smerdou

William Smiles

Richard W.P. Smith

David Smith

Pavel Solopov

Minsoo Song

Nagasamy Soumittra

Mário Sousa

Graça Soveral

Erdi Sozen

Riccardo Spizzo

Michael Spurway

Sree Rama Chaitanya Sridhara

Piyarat Srisawang

Alexis Stamatikos

Chloe Stanton

Jonathan A. Stiber

Dovile Stravinskiene

Sabine Strehl

Emanuel E. Strehler

Rocío Suárez‐Sánchez

Mirza Subhan

Ganesh Subramani

Colleen Sweeney

Agata Święciło

Csaba Szabo

Andras Szollosi

Paul Taghert

Yuichiro Takagi

Szabolcs Takats

David Talens‐Perales

Annadurai Thangaraj

Kavitha Thirumurugan

Shiyi Tian

Henning Tidow

Smilja Todorovic

Tsukasa Tominari

Scott Tomlins

Renata Torres da Costa

Jorg Tost

Ray Truant

Masahiro Ueda

Elizabeth Vafiadaki

Cristian Valdes

Sarah van Veen

Luca Vanella

Sakari Vanharanta

Mark Varvares

Silvia Vega Rubin de Celis

Guillermo Velasco

Digvijay Verma

Marçal Vilar

Jose Luis Vilas

Ly Villo

Susanne Voelkel

Tobias von der Haar

Feng Wang

Fiona E. Watt

Clinton Webb

Ian Webb

Sebastian Westenhoff

Iain B. H. Wilson

Gerlof Sebastiaan Winkler

Kenneth Wong

Lei Xi

Hui Xiao

Bang Xiao

Wei Xie

Wen‐Yong Xiong

Na Xu

Sean A. Yamada‐Hunter

Chuan Yan

Ryo Yanagiya

Cheng Yang

Samantha Yeligar

Haidi Yin

Jodi Young

Tracy Young‐Pearse

Manda Yu

Dietmar M. W. Zaiss

Markella Zannikou

Lucie Zemanova

Jun Zhang

Bingsen Zhang

Xiaofei Zhang

Tong Zhang

Bo Zhou

